# CD8 Memory Cells Develop Unique DNA Repair Mechanisms Favoring Productive Division

**DOI:** 10.1371/journal.pone.0140849

**Published:** 2015-10-20

**Authors:** Alessia Galgano, Aleksandr Barinov, Florence Vasseur, Jean-Pierre de Villartay, Benedita Rocha

**Affiliations:** 1 INSERM, U1020, CNRS, UMR 8253, Medical Faculty Paris Descartes, Université Paris Descartes Sorbonne Paris Cité, Paris, France; 2 INSERM, UMR 1163, Institut Imagine, Hôpital Necker-Enfants Malades, Paris, France; Tulane University Health Sciences Center, UNITED STATES

## Abstract

Immune responses are efficient because the rare antigen-specific naïve cells are able to proliferate extensively and accumulate upon antigen stimulation. Moreover, differentiation into memory cells actually increases T cell accumulation, indicating improved productive division in secondary immune responses. These properties raise an important paradox: how T cells may survive the DNA lesions necessarily induced during their extensive division without undergoing transformation. We here present the first data addressing the DNA damage responses (DDRs) of CD8 T cells *in vivo* during exponential expansion in primary and secondary responses in mice. We show that during exponential division CD8 T cells engage unique DDRs, which are not present in other exponentially dividing cells, in T lymphocytes after UV or X irradiation or in non-metastatic tumor cells. While in other cell types a single DDR pathway is affected, all DDR pathways and cell cycle checkpoints are affected in dividing CD8 T cells. All DDR pathways collapse in secondary responses in the absence of CD4 help. CD8 T cells are driven to compulsive suicidal divisions preventing the propagation of DNA lesions. In contrast, in the presence of CD4 help all the DDR pathways are up regulated, resembling those present in metastatic tumors. However, this up regulation is present only during the expansion phase; i.e., their dependence on antigen stimulation prevents CD8 transformation. These results explain how CD8 T cells maintain genome integrity in spite of their extensive division, and highlight the fundamental role of DDRs in the efficiency of CD8 immune responses.

## Introduction

The capacity of T cells to recognize all antigens relies on their very diverse naïve T cell repertoire. Cells specific for each antigen are therefore quite rare, but immune responses are efficient because lymphocytes have a remarkable capacity to expand. Indeed, each individual CD4 T cell was shown to be able to generate up to a 10^14^ progeny [1]. To deal efficiently with rapidly dividing pathogens, lymphocyte division and accumulation is also rapid, CD8 T cell numbers increasing up to 10,000 fold in a few days after immunization [2]. These division properties pose an important paradox. Multiple divisions should increase DNA breaks, which must be repaired to allow T cell survival and prevent transformation. However, the rapid T cell accumulation during immune responses is only possible because T lymphocytes’ cycle time is of short duration [3], reducing the time available for efficient repair. It is not known how T cells address this paradox. The responses to DNA damage (DDRs) were never evaluated during immune responses. However, our previous analyses of CD8’s progression through divisions indicate that the rate of cell death during division determines the amplitude of each response: CD8 accumulation collapses in the absence of help [4], while in the presence of CD4 help the higher amplitude of secondary responses is due to a much-increased cell survival during division [3]. Therefore, we here evaluated if CD8 T cells in secondary responses could have improved DDRs and if such improvement would depend of CD4 help.

In general, DNA lesions may be double strand (DSB) or single strand breaks (SSB), the former believed to be dominant during cell division [5]. In both cases, DNA damage is recognized by the same genome sensing histone H2AX. DSBs recruit the MRN complex, which holds the DNA ends together stabilizing the break, and recruits the ATM kinase [6]. This kinase has multiple fundamental roles. It propagates DSB-induced changes in the chromatin structure, phosphorylates H2AX and subsequently Artemis. ATM also phosphorylates TP53BP1, (which concentrates ATM at the DSB and amplifies the effects of the MRN complex), the BRCA1 mediator, CHK2 transducer and binds to TP53, which leads to cell cycle arrest. Once the cell cycle is arrested, DNA repair molecules are recruited to the DNA damaged sites [7,8].

Cells have evolved two main pathways to repair DSBs: non-homologous end joining (NHEJ) and homologous recombination (HR) [9]. NHEJ is believed to be error prone, since the two broken DNA ends are rejoined directly (without the use of a “DNA repair template”) leading to small deletions of the DNA sequence [9]. In contrast, during HR, DSBs are corrected in an error-free manner by using the undamaged sister chromatid as the repair template [9]. However, these pathways are not fully independent, efficient HR requiring efficient NHEJ [10].

SSBs recruit the 9-1-1 complex, rather than MRN [7]. It is still unclear how the different members of this complex interact and are successively recruited to the damaged site, but ATR associated with chromatin phosphorylates RAD17, is necessary to trigger the response. The damage signal is transduced by CHK1 that phosphorylates CDC25 proteins. This phosphorylation inactivates CDC25 proteins, leading to cell cycle arrest [7].

Several mechanisms may be used to repair SSBs. In the base excision repair (BER), base damages may be corrected by different DNA glycosylases and the APEX1 endonuclease removes the damaged bases. Nucleotide excision repair (NER) removes bulky DNA lesions. Mismatch repair (MMR) solves mismatched bases (A-G or C-T). Lastly, in direct DNA repair, the only known acting enzyme is MGMT (methyl-guanine methyltransferase), which removes the O^6^-metyl group from O^6^-methyl guanine [8].

In this study, we evaluated how CD8 T cells cope with the DNA lesions during immune responses in the absence or presence of CD4 help. By using pathway-focused PCR array-based expression profiling, we here show that DDR modifications are restricted to the exponential phases of the immune responses. While DDRs are possible during primary response, they fail in secondary responses in the absence of CD4 help. Then, CD8 T cells cannot sense DNA damage and all the mechanisms of repair fail. Moreover, they cannot stop cycling to repair i.e., they undergo compulsive suicidal divisions. The overall DDR responsible for this failure are not present in other exponentially dividing cells, differ from those induced in T cells after mitogen stimulation, by X or UV irradiation and from those reported in non-metastatic tumors. In contrast to CD8 responses in the absence of CD4 help, when CD4 help is present, DNA damage sensing is adequate and all DDR pathways are up regulated ensuring adequate cell survival during division, resembling the DDRs gene expression reported in metastatic tumors. However, transformation does not occur, since modifications of DDR are restricted to CD8 exponential growth phases, i.e., require antigen in order to be induced and maintained. It must be noted that since we studied CD8 T cells “*ex vivo*” the number of cells we could recover was very low. Therefore, in this study we could only evaluated mRNA levels while the corresponding protein levels may be spatial-temporally different during DDR, due to reversible post-translational modifications [11]. Emerging evidence indicates that DDR actually involves complex and yet unclear networks of different factors, which are influenced by post-transcriptional and post-translational modifications affecting nearly all DNA repair factors [12]. These modifications may reversibly/dynamically alter the levels of acting/activating proteins at each step of the DNA repair [13]. Our data, however, further shows that besides these post-translation modifications, down- and up-regulation of the expression of DNA repair genes also occur in DDR responses.

## Results and Discussion

### Experimental strategy

To study possible modifications of DDRs during CD8 primary and secondary immune responses *in vivo* we excluded differences in TCR specificity and initial T cell numbers in both responses. We studied the same monoclonal (Mo) population of CD8 T cells, expressing a transgenic TCR (TCR-Tg) specific for the male antigen [14]. Naïve cells were recovered from Rag-2^-/-^TCR-Tg female mice. CD8 T cells at different stages of the immune responses were obtained after adoptive transfer of these naïve cells and their immunization with male bone marrow (BM), in the absence or presence of CD4 T cells from untreated C57/Bl6 female mice. Since the kinetics of both primary and secondary responses was well established in this system [3] we could sort CD44^+^ CD8 Tg cells from the spleen in the primary responses during the phases of exponential growth, contraction and memory and in the exponential phase of the secondary responses. DDRs were evaluated by pathway-based gene expression profiling in two PCR arrays including 127 genes with established roles in DNA damage signaling, cell cycle arrest, DNA repair and induction of apoptosis.

### In the absence of CD4 help, DNA repair is possible in the primary CD8 response, but is compromised in the secondary CD8 response

**Fig 1:**
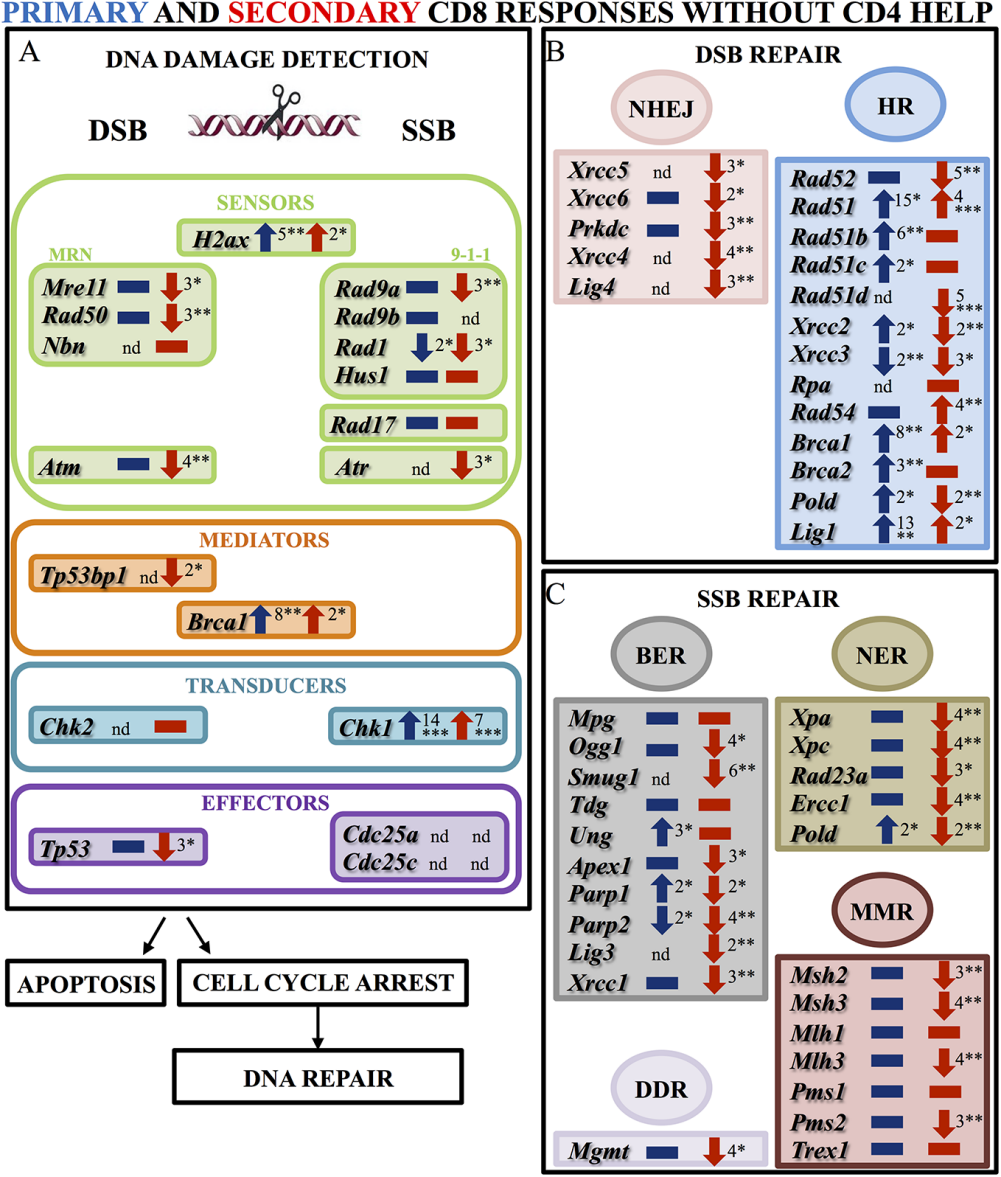
DNA damage responses in CD8 T cells in the absence of CD4 help. Results show the expression of genes involved in: (**A)** DNA damage checkpoints (sensors, mediators, transducers and effectors of double and single strand break (DSB; SSB)); (**B)** DSB repair mechanisms (non homologous end-joining (NHEJ) and homologous recombination (HR)); (**C)** SSB repair mechanisms (base excision repair (BER), nucleotide excision repair (NER), mismatch repair (MMR) and direct DNA repair (DDR)) were analyzed during primary and secondary CD8 responses generated in the absence of CD4 cells using mouse DNA damage signaling pathway and the DNA repair RT^2^ profiler PCR arrays (Qiagen). For each experiment, CD8 T cells were pooled from 4 mice in primary responses and 4–6 in secondary responses. The average expression level of unmodified housekeeping genes selected among the five included was used for normalization. Results are expressed as fold change relative to reference samples (naïve cells in primary responses; memory cells in secondary responses) and represent the average of 3 to 4 independent experiments. Primary responses are shown in blue; secondary responses in red. Up and down arrows indicate up and down regulation respectively; rectangles indicate no significant changes in gene expression. Asterisks indicate statistical significance: *, p < 0,05; **, p ≤ 0,01; ***, p ≤ 0,001.

We first compared the expression of genes involved in DDR in the primary and secondary CD8 responses in the absence of CD4 help, as compared respectively to naïve or memory cells, the latter recovered from 4 to 6 months after priming (Fig 1, S1 Table).

In primary responses, the expression of *H2ax* coding for the histone guardian of the genome was 5-fold increased, reflecting that DNA damage occurs [15]. In general, CD8 T cells could detect both DSB and SSB, since the transcript levels of the different components of the MRN and the 9-1-1 complexes, as well as *Atm* and *Atr* were unmodified. The *Brca1* mediator, (which functions in both the DSB and SSB pathways [7] was much up-regulated as well as the *Chk1* transducer, which was 14-fold increased. Lastly, *Tp53* expression was maintained (Fig 1A). Overall, these results indicate that CD8 T cells in primary responses maintain an adequate sensing of DNA breaks and are able to undergo cell cycle arrest to repair these damages.

This repair is likely done preferentially by HR rather than by NHEJ. While the expression of genes involved in NHEJ was not modified, the majority of the genes involved in HR were up regulated (Fig 1B). In particular, the expression of *Rad51*, which plays a fundamental role in HR [16], was 15-fold up-regulated. The 8-fold up-regulation of *Brca1* expression, a mediator which plays a central role in HR [17], also supports that HR might be preferred to NHEJ. Our study revealed no significant variation in the diverse SSB repair pathways: in general, gene levels were unmodified, and for the only gene for which down-regulation was found (*Parp2*), the expression of other member of the same gene family was up-regulated (*Parp1*), thus indicating that SSBs could be correctly resolved (Fig 1C). Overall, this data indicates that during primary responses both SSB and DSB can be sensed and repaired upon cell cycle arrest.

These results contrast with the expression of these genes in secondary responses in the absence of CD4 T cells (Fig 1, S1 Table). While *H2ax*, which codes for the histone guardian of the genome was yet up regulated, the detection of DNA damage was much impaired (Fig 1A). Most of the components of MRN and 9-1-1 complexes as well as *Atm* and *Atr* were down regulated, indicating the DNA damage sensing machinery was deficient. Moreover, *Tp53bp1* that concentrates ATM at the DSB sites and amplifies the effects of the MRN complex [18] was 2-fold reduced. *Tp53* expression was also 3-fold reduced, what should hinder cell cycle arrest (Fig 1A). Lastly, the expression of the genes involved in DNA repair was also compromised (Fig 1B and 1C). The genes required for NHEJ were down regulated at day 4, and declined further at day 6 (S1 Table). Genes involved in HR were expressed at lower levels than in the primary response. Among these, the expression of *Rad52* that binds broken DNA ends [9] was 5-fold reduced. Although this defect may be compensated by cooperating RAD51 activity [19], the down regulation of *Prkdc*, essential for HR [10] (Fig 1B), should compromise HR efficiency. Lastly, SSB repair was also compromised since the majority of the genes involved in this repair were less expressed (Fig 1C).

Overall these results justify the major defects in cell expansion observed in secondary responses in the absence of CD4 help [4]: DNA damage sensing machinery in these CD8 T cells seems to be deficient, resulting in restricted repair of DNA DSB breaks during division. CD8 capacity to stop dividing is also compromised. Therefore, CD8 T cells should continue dividing and accumulate DNA breaks until the collapse of the replicating fork, i.e., they should undergo compulsive suicidal divisions.

We also studied the expression of DDR genes in the contraction and in the memory phases both in the absence and in the presence of CD4 cells. We found that expression of the majority of genes involved in DDRs were not modified during the contraction phase (S2 Table) indicating that the cell death then occurring is not due to a failure in DDRs. However, memory cells generated in the absence of CD4 help showed increased expression of *H2ax*, suggesting the persistence of DNA breaks, and the down-regulation of the expression of *Atm* and *Atr*, in DSB and SSB sensing, and *Prkdc* in NHEJ (S3 Table). We conclude that modifications of the DDRs are mostly restricted to the exponential phase of the immune responses, when cells are actively dividing. However, the presence of CD4 help during primary responses appears to be required for the generation of memory cells with intact DDRs machinery.

We also studied the expression of other genes with secondary roles in DDR (S4 Table) and those known to be involved in DDR, but with less defined function (S5 Table). The most important modification was a 35-fold up regulation of *Fancg* during secondary responses in the absence of CD4 help. This is one of the seven Fancony anemia (FA) genes coding for proteins that bind chromatin or localize to sites of DNA damage [20].

### CD8 T cells’ DDRs become fully efficient in secondary responses in the presence of CD4 help

**Fig 2:**
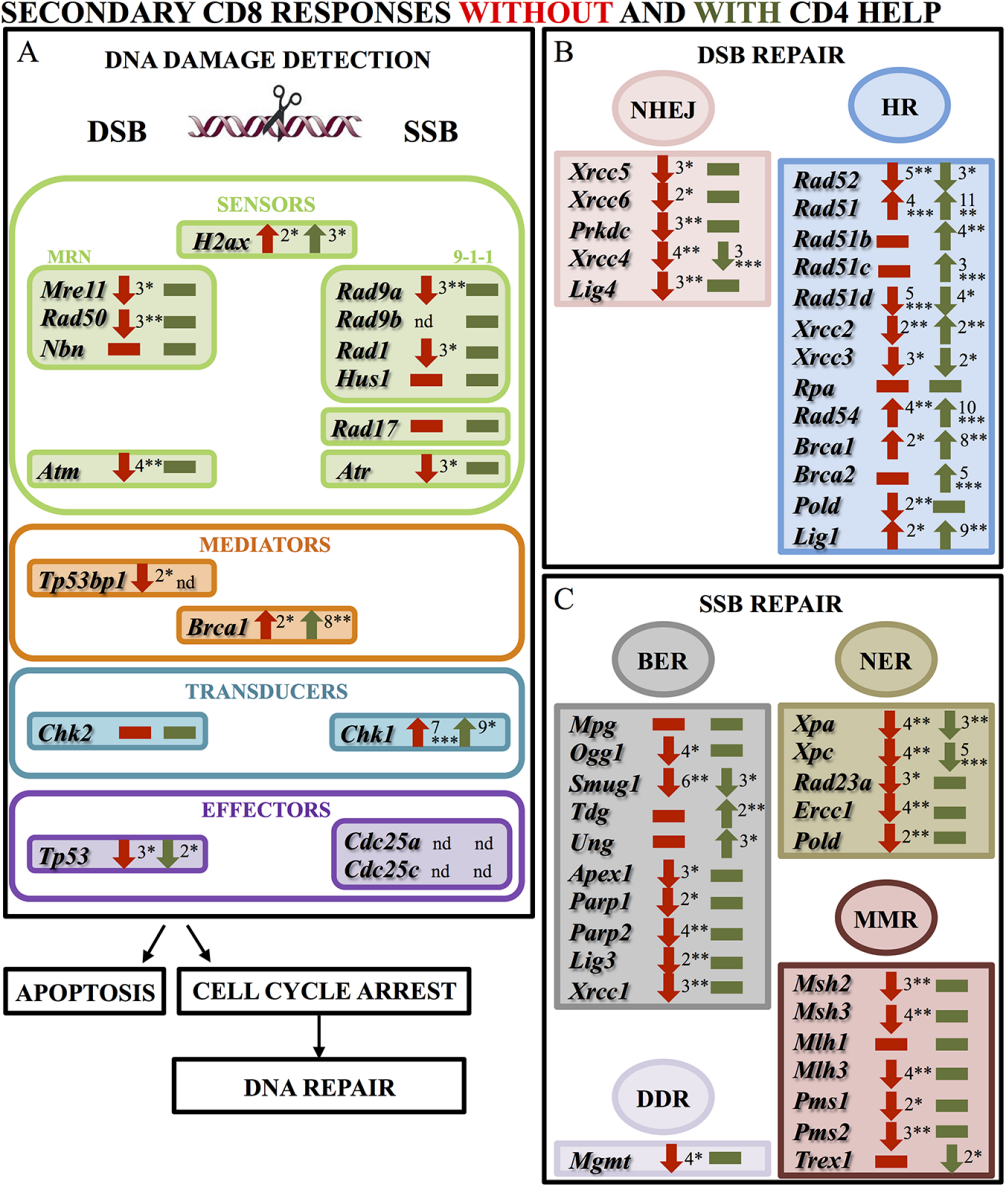
Comparison of DNA damage responses in CD8 T cell secondary proliferation in the absence and presence of CD4 T cells. Results show the expression of genes involved in: (**A)** DNA damage checkpoints; (**B)** DSB repair and (**C)** SSB repair, analyzed during secondary CD8 responses generated in the absence and presence of CD4 cells. Analysis was performed using mouse DNA damage signaling pathway and DNA repair RT^2^ profiler PCR arrays (Qiagen). CD8 T cells were pooled from 4–6 mice for each experiment in absence of CD4s and 3 mice in presence of CD4s. Results were normalized as described in Fig 1, expressed as fold change relative to memory cells. They represent the average of 4 to 5 independent experiments. Responses without CD4s are shown in red; with CD4s in green. Up and down arrows indicate up and down regulation, respectively; rectangles indicate no significant changes in gene expression. Asterisks indicate statistical significance: *, p < 0,05; **, p ≤ 0,01; ***, p ≤ 0,001.

While the presence of CD4 T cells does not improve DDR in primary responses, it has a major role in secondary immunizations (Fig 2, S6 Table). The expression of the intrinsic sensor *H2ax* was 3-fold increased. The expression of DNA damage sensor genes were unmodified, indicating that DNA damage is properly sensed in these dividing memory cells (Fig 2A). As found in the other CD8 responses, HR may also be the preferential mechanism involved in repair. The expression of the majority of the genes involved in this pathway was up regulated, showing increased expression levels when compared to secondary responses in the absence of CD4 T cells. Although the expression of *Rad52*, *Rad51d* and *Xrcc3* were slightly reduced, they were expressed at higher levels than in secondary responses in the absence of CD4 help. The very high up-regulation of their respective analogues should compensate this slight reduction. NHEJ mechanisms were not substantially affected, as *Xrcc4* was the only gene to show a 3-fold reduction (Fig 2B). The diverse mechanisms used to repair damaged bases will likely function efficiently since most of the genes involved in this repair are unmodified, with few exceptions: BER-associated DNA glycosylase-coding *Smug1*, whose reduced expression might be replaced by the others showing unmodified levels; *Xpa* and *Xpc* sensors in NER, which may impair damage sensing, and *Trex1* coding for an endonuclease acting in MMR (Fig 2C).

Overall, these results show that while DNA repair is significantly compromised in secondary responses in the absence of CD4 cells, the repair mechanisms should function efficiently when CD4s are present. It must be noted that we still observed a 2-fold down-regulation of *Tp53* (Fig 2A), suggesting that despite the efficiency of upstream checkpoints, cell cycle might not be arrested. Since DNA repair mechanisms may yet function in absence of DNA damage-induced cell cycle arrest [7], the down-regulation of *Tp53* may not affect repair.

### The DNA damage responses and the regulation of cell cycle checkpoints found in CD8 immune response are not shared by other exponentially dividing cells

To determine whether the DDR expression patterns observed during CD8 exponential growth were just a consequence of cell division, or rather reflect the onset of a lymphoid specific DDR program, we evaluated DDRs in other non-transformed, rapidly dividing cells. We studied primary mouse embryonic fibroblasts (PMEF), which are not related to hematopoietic, lymphoid or stem cells, and proliferate vigorously *in vitro*. Comparison of resting to exponential growing cells showed little differences in DDRs’ gene expression profiles. In the expansion phase *Lig4* and *Ung* were up-regulated, while *Xrcc5* was down regulated, but these modifications were lower than 2-fold (S7 Table). These results show that other exponentially dividing cells do not have the major modifications of DDR found in CD8 immune responses.

Therefore, this pathway-specific PCR-based approach to study DDRs in CD8 T cells and other non-related dividing cells show a unique pattern of DDRs in CD8 T cells, which does not overlap with those detected in other dividing cells. Without CD4 help, DDRs functioned properly during primary, but were severely impaired in secondary responses. The presence of CD4 T cells did not improve DDRs in the primary, but improved considerably DDRs in the secondary response. Overall these results provide an explanation to the collapse of CD8 accumulation during secondary responses generated in the absence of CD4 help, and the major amplitude of CD8s’ secondary responses when CD4 help is available [4].

We found that DDRs were mostly active during the expansion phase. DDRs were absent in the contraction phase, indicating that the cell death then occurring is not due to deficient DDR. These results demonstrate that DDRs are restricted to fast dividing CD8 T cells and signify that the presence of antigen is most probably required for their induction/maintenance. This requirement is likely fundamental to prevent T cell transformation, as discussed below.

We found common features to all CD8 dividing cells. Thus, *H2ax* coding for the histone guardian of the genome was always up-regulated, indicating that sensing of genome damage always occurred, and was independent of the subsequent events required for DDR. The overall gene expression patterns indicate that the repair was always oriented to HR. In all conditions, we found an up-regulation of *Brca1*, favoring HR [21] and the genes involved in HR were generally up regulated. Thus, CD8 division aims to preserve DNA integrity, since HR preserves genome integrity, while NHEJ functions primarily to maintain cell viability [9].

Other features varied in different responses. The severe dysfunction of DDRs found in secondary responses in the absence of CD4 T cells affected all DDR pathways. We found a major deficiency in the components of both MRN and 9-1-1 complexes, leading to the down-regulation of both *Atm* and *Atr* and associated complexes. The down-regulation of these genes affected downstream components of cell cycle checkpoints, such as *Tp53bp1* mediator and *Tp53* effector. Indeed, it was shown that the ATM deficiency affects TP53 activation, since TP53 is mainly activated by ATM phosphorylation [22]. Moreover, *Tp53* down-regulation promotes genome instability and deregulation of T cells apoptosis [23]. In addition, defects in TP53 activation have deleterious effects on NHEJ, since NHEJ defects are amplified and therefore DSBs are replicated what could lead to important modifications in the genome [24]. These conditions should be further aggravated by the reduced expression TP53BP1 [25]. These modifications, together with the down-regulation of the genes involved in NHEJ should compromise HR, in spite of the up-regulation of the expression of some genes involved in this repair pathway. Indeed, a recent study revealed that NHEJ efficiency and in particular DNA-dependent protein kinase catalytic subunit (DNA PKcs) activity, is essential for HR to occur [10]. Lastly, SSB repair was also compromised, since the expression of the majority of genes involved in BER, NER, MMR or DDR were down regulated. Therefore, in these conditions (impairment of all DDR pathways, together with decreased *Tp53* levels hindering cell cycle arrest), DNA breaks would continue to accumulate leading to the collapse of the replication fork and survival endangerment, i.e., CD8 T cells would undergo compulsive suicidal divisions.

In contrast, in presence of CD4 T help, DDR appears to be very efficient. The MRN, the 9-1-1 complexes, *Atm* and *Atr* expression are at normal levels. *Brca1* levels are much up regulated, orienting repair to HR. The integrity of NHEJ pathway, as well as the very high expression levels of genes involved in HR should ensure efficient HR repair. Lastly SSB should not be compromised. It must be noted that even in these cells *Tp53* remains down regulated, what could compromise cell cycle arrest. Overall, these results demonstrate that once activated, CD8 T cells may not stop dividing. In the absence of CD4 help this division leads necessarily to cell death. When CD4 T cells are present CD8 T cells repair DNA efficiently and thus survive.

The question arises about how much the different DDRs found in CD8 responses are shared by other dividing cell types. In exponentially dividing PMEF, we only noticed a less than two fold increase in few genes involved in BER, attesting that CD8s’ DDRs is not common to all exponentially diving cells. The DDRs found in CD8 immune responses also differ from those described in lymphocytes after mitogen stimulation, or upon exposure to ionizing and non-ionizing radiations, which constitute exogenous sources of DNA damage. The expression of genes involved in DDRs do not differ between resting and mitogen-stimulated cells, the majority of the genes with known roles in DDRs showing constant expression levels and those which were up-regulated being also involved in DNA replication [26]. Upon ionizing radiations, no changes at the transcriptional level of *Atm* were observed [27]. The DDRs induced after exposing of resting or mitogen-stimulated lymphocytes to ionizing irradiation also have different characteristics: it was reported that DNA repair capacity is not increased after high dose radiation exposure [28], thus indicating that DDR genes are already present in sufficient amounts to properly repair radiation-induced DNA damages. NHEJ and HR mechanisms were not, or were only slightly affected by irradiation. Increased expression levels were only observed for most genes acting in BER and NER (reviewed in [29]). Overall, these results indicate that DDRs induced after exposition of resting or mitogen-stimulated CD8 T cells to ionizing and non-ionizing radiations are different from that observed during CD8 immune responses.

The DDRs found in CD8 responses also differ from those found in non-metastatic tumors. The molecular origin of carcinogenesis is the accumulation of DNA repair defects, which promotes genomic instability [30]. Therefore modifications of DDRs were extensively studied in tumor cells, different types of tumors having different types of DDR impairment. Patients with primary immune-deficiencies caused by inherited alterations in genes involved in DDRs are known to have an increased incidence of cancers [30,31]. Germ-line mutations in genes acting in DNA damage sensing and repair are responsible for many familial cancer syndromes, such as hereditary non-polyposis colorectal cancer (HNPCC) which is due to MMR mutations, BRCA-deficient breast and ovarian, endometrium and gastric cancers [32]. Defects in NHEJ pathway are at the origin of the development of myeloid and lymphoblastic leukemia and lymphomas [33], whereas over-expression of NHEJ genes is responsible for chemotherapy resistance in B-chronic lymphocytic leukemia (B-CCL) [34]. Mutations in genes acting in DSB sensing and recruitment of HR machinery to DSB site are found in multiple myeloma [35]. However, there are major differences between the compromised DDRs found in non-metastatic tumors and those found in CD8 cells dividing in the absence of CD4 help. In each different tumor DDR defects affect a single repair pathway, the pathway affected differing in each tumor. Therefore, although their DDR defects predispose tumor cells to apoptosis, tumor cells may yet use alternative pathways of repair allowing their survival. This survival will allow propagation and amplification of DNA modifications, as it is known to occur during tumor growth. Instead, in the compromised DDR found in immune responses, all different repair pathways are affected. Therefore, all dividing cells will necessarily die, preventing the unwanted propagation of genome modifications.

While impairment of repair mechanisms is required to induce malignant transformation, improved DDR efficiency is needed for tumor progression and metastasis. Thus, metastatic tumors show an over-expression of genes involved in HR pathway [32], as we find in CD8 secondary responses in the presence of CD4 help. However, our overall data allows understanding how the CD8 extensive division during secondary responses in the presence of CD4 help does not result in lymphoid transformation. Improved DDR efficiency was only found in the exponential growth phase, declining when the antigen is eliminated. Therefore, the immune system developed efficient methods to guarantee timely cell accumulation during immune responses, while preventing T cell transformation in spite of the CD8 extensive division.

## Materials and Methods

### Mice and immunization procedures

Mice were 6–8 week old C57/Bl6, bred at the Center for the Development of Advanced Animal Experimentation (Orleans, France). Immune responses were performed as described previously [3]. Briefly, sub-lethally irradiated (400Rad) female Rag-2^-/-^ mice were injected i.v. with 4.5x10^6^ female and 0.5x10^6^ male bone-marrow (BM) cells from CD3ε^-/-^ mice. Three days later, reconstituted mice received i.v. 0.5x10^6^ CD8 naïve cells T from female Rag-2^-/-^ mice transgenic for a TCR αβ specific to the HY male antigen [14], either alone, or together with the same number of purified CD4 T cells from untreated C57/Bl6 female mice. Purified CD4 T cell populations were obtained after depletion of non-CD4 T cells by magnetic sorting using Dynabeads (Invitrogen Dynal AS) [3]. Secondary responses were performed using the same protocol but the injected CD8 Tg cells were memory cells, sorted from the immunized mice described above, 4–6 months after priming. Since the kinetics of these CD8 responses was described in detail previously [3], we were able to recover TCR Tg cells from the spleen at well known time points of these responses. In each experiment the spleen cell suspensions were pooled from 4–5 mice, and the CD8 TCR Tg cells were sorted from: (i) female TCR-Tg mice (Naïve); and at different days after immunization: during the primary response, (ii) at the exponential growth phase (day 6) (iii) at the contraction phase (day 19) (iv) at the memory phase (4–6 months after priming); in the secondary response in the absence of CD4 T cells: during the exponential growth phase (v) at day 4 and (vi) day 6 after boosting; in the secondary response with CD4, at the exponential growth phase (day 4). Mice were sacrificed by carbon dioxide asphyxia. Animal experimental procedures were designed to minimize animal suffering, were approved by the French University Animals Ethics Committee and conducted according to the institutional guidelines of the European Community.

### Antibodies and immunofluorescence analysis

Monoclonal antibodies used for flow cytometry and cell sorting were: PE-labeled rat anti mouse anti-CD44 (IM7 clone from BD Pharmingen). FITC-labeled rat anti mouse anti-CD8β (H35-172, BD Pharmingen) and biotin-labeled anti-T3.70 (anti-TCRα Tg), were conjugated in our laboratory. The biotinylated antibody was revealed with Streptavidine Allophycocyanin (APC) (BD Pharmingen). Cells were analyzed on a FACSCanto II, sorted on a FACSAria, and data analysis was performed using Diva software (Beckton Dickinson, Franklin Lakes, NJ, USA). The final antibody concentrations were used according to the manufacturer’s instructions.

### PMEF cell cultures

Primary mouse embryonic fibroblast (PMEF) cells were generated by J.P. de Villartay. These cells were cultured in Petri Dishes at 37°C in a humidified 5% CO_2_ incubator in high glucose Dulbecco’s modified Eagle’s Medium supplemented with GlutaMAX, 1 mM sodium pyruvate, 10% Fetal Calf Serum and 1% penicillin-streptomycin (Gibco, Carlsbad, CA, USA). Cells were recovered at the exponential growth and confluent phases.

### Pathway-specific PCR arrays

Total RNA from CD8 TCR Tg or PMEF cells was isolated using RNeasy Mini kit (Qiagen, Hilden, Germany) and quantified with a NanoDrop2000 device (Thermo Scientific, Waltham, MA, USA). 30ng and 50ng total RNA was used from respectively CD8 TCR Tg and PMEF cells. Gene expression analysis was performed using arrays for mouse DNA damage signaling pathways (Cat. No. PAMM-029ZC) and DNA repair (Cat. No. PAMM-042ZC) RT^2^ profiler PCR arrays (Qiagen, Hilden, Germany). The two arrays contained 127 unique genes with known roles in DDR. cDNA synthesis, pre-amplification of cDNA targets and real-time PCR were performed according to the manufacturer’s instructions. Data analysis was performed using the Excel-based PCR array data analysis template provided. Gene expression was normalized to the average of unmodified housekeeping genes (selected among the five included) and fold differences were quantified according to the 2^–ΔΔCt^ method. Results were expressed as fold change relative to reference samples (naïve cells in primary responses; memory cells in secondary responses). Fold change values less than 1 indicated down-regulation and were expressed as -1/fold difference (i.e. 2^–ΔΔCt^ = 0.5 would be expressed as 2-fold down-regulation or a fold change of -2). Statistical analysis was performed using Student’s t-test and statistical significance was set at p ≤ 0.05. Data shown represent the average of three to five independent experiments.

### Real time PCR analysis

Statistically significant differentially expressed genes were further validated by SYBR Green real-time PCR (Applied Biosystems, Carlsbad, CA, USA), using the same RNA samples previously used for PCR arrays. RNA was first amplified using Message AmpII aRNA amplification kit (Ambion Carlsbad, CA, USA) according to the manufacturer’s instructions and then reverse-transcribed using random hexamers (Applied Biosystems Carlsbad, CA, USA). Primer sequences were designed with Primer3 on line software (http://primer3.ut.ee/) and synthesized by Eurogentec-France. Data analysis was performed as described above.

## Supporting Information

S1 Table Primary and secondary CD8 responses without CD4 help. (PDF)

S2 Table Contraction phase of primary CD8 responses without and with CD4 help. (PDF)

S3 Table Memory phase without and with CD4 help. (PDF)

S4 Table Additional modified genes with secondary roles in DNA damage response. (PDF)

S5 Table Additional modified genes with less defined function in DNA damage response. (PDF)

S6 Table Secondary CD8 responses without and with CD4 help. (PDF)

S7 Table Exponential growing *vs*. resting PMEF cells. (PDF)
