# Supplementary material for: CD8 Memory Cells Develop Unique DNA Repair Mechanisms Favoring Productive Division
Source: PLoS One. 2015 Oct 20;10(10):e0140849. doi: 10.1371/journal.pone.0140849 (PMC4613136; doi:10.1371/journal.pone.0140849)
Supplement: S1 Table — (PDF) [file pone.0140849.s001.pdf]

Table S1. Primary and secondary CD8 responses without CD4 help

			Gene	Primary response		Secondary response			
				d6		d4		d6	
				Fold change	p value	Fold change	p value	Fold change	p value
DSB DETECTION	SENSORS		***H2ax***	**4,7**	**0,003**	**1,7**	**0,02**	**2,2**	**0,006**
DSB DETECTION	SENSORS	MRN	***Mre11***	-1,1	0,72	**-2,7**	**0,02**	-1,8	0,1
DSB DETECTION	SENSORS	MRN	***Rad50***	1,1	0,47	**-3,3**	**0,01**	-2,1	0,05
DSB DETECTION	SENSORS	MRN	***Nbn***	nd		-1,3	0,64	1,4	0,39
DSB DETECTION	SENSORS		***Atm***	-1,2	0,65	**-4,2**	**0,004**	**-2,9**	**0,04**
DSB DETECTION	MEDIATORS		***Tp53bp1***	nd		**-2,0**	**0,02**	**-1,9**	**0,02**
DSB DETECTION	MEDIATORS		***Brca1***	**7,6**	**0,003**	**2,2**	**0,03**	**4,6**	**0,00007**
DSB DETECTION	TRANSDUCER		***Chk2***	nd		1,1	0,67	4,5	0,07
DSB DETECTION	EFFECTOR		***Tp53***	-1,4	0,12	**-2,7**	**0,03**	**-1,9**	**0,008**
SSB DETECTION	SENSORS		***H2ax***	**4,7**	**0,003**	**1,7**	**0,02**	**2,2**	**0,006**
SSB DETECTION	SENSORS	9-1-1	***Rad9a***	-1,6	0,25	**-2,7**	**0,01**	**-2,9**	**0,00007**
SSB DETECTION	SENSORS	9-1-1	***Rad9b***	-1,7	0,1	nd		nd	
SSB DETECTION	SENSORS	9-1-1	***Rad1***	**-1,8**	**0,02**	**-2,5**	**0,03**	**-2,9**	**0,00002**
SSB DETECTION	SENSORS	9-1-1	***Hus1***	1,4	0,29	-1,8	0,1	-1,1	0,95
SSB DETECTION	SENSORS		***Rad17***	-1,3	0,11	-3,1	0,08	**-1,9**	**0,001**
SSB DETECTION	SENSORS		***Atr***	nd		**-3,4**	**0,04**	-3,1	0,08
SSB DETECTION	MEDIATOR		***Brca1***	**7,6**	**0,003**	**2,2**	**0,03**	**4,6**	**0,00007**
SSB DETECTION	TRANSDUCER		***Chk1***	**14,3**	**0,001**	**6,6**	**0,001**	**17,3**	**0,01**
DSB REPAIR		NHEJ	***Xrcc5***	nd		**-2,7**	**0,02**	**-3,5**	**0,012**
DSB REPAIR		NHEJ	***Xrcc6***	1,4	0,24	**-2,2**	**0,02**	**-2,2**	**0,06**
DSB REPAIR		NHEJ	***Prkdc***	1,4	0,30	**-3,0**	**0,01**	**-2,8**	**0,02**
DSB REPAIR		NHEJ	***Xrcc4***	nd		**-4,1**	**0,003**	**-5,7**	**0,009**
DSB REPAIR		NHEJ	***Lig4***	nd		**-2,9**	**0,009**	**-2,0**	**0,03**
DSB REPAIR		HR	***Rad52***	-1,0	0,95	**-4,8**	**0,009**	**-4,4**	**0,02**
DSB REPAIR		HR	***Rad51***	**14,5**	**0,03**	**4,2**	**0,0002**	**16,8**	**0,04**
DSB REPAIR		HR	***Rad51b***	**6,4**	**0,008**	1,3	0,65	**2,4**	**0,007**
DSB REPAIR		HR	***Rad51c***	**2,3**	**0,03**	1,7	0,09	**7,2**	**0,00005**
DSB REPAIR		HR	***Rad51d***	nd		**-5,4**	**0,001**	**-3,2**	**0,02**
DSB REPAIR		HR	***Xrcc2***	**1,9**	**0,03**	**-1,8**	**0,01**	-1,2	0,32
DSB REPAIR		HR	***Xrcc3***	**-1,9**	**0,003**	**-3,2**	**0,02**	**-2,9**	**0,04**
DSB REPAIR		HR	***Rpa***	nd		-2,2	0,05	-1,4	0,25
DSB REPAIR		HR	***Rad54***	1,0	0,67	**3,5**	**0,003**	**6,8**	**0,0005**
DSB REPAIR		HR	***Brca2***	**3,3**	**0,01**	-1,1	0,8	1,9	0,1
DSB REPAIR		HR	***Pold***	**1,7**	**0,03**	**-2,2**	**0,01**	-1,3	0,31
DSB REPAIR		HR	***Lig1***	**12,6**	**0,002**	**1,9**	**0,046**	**5,4**	**0,0006**
REPAIR		BER	***Mpg***	1,7	0,17	-2,0	0,06	-2,1	0,05
REPAIR		BER	***Ogg1***	1,2	0,44	**-3,7**	**0,02**	-2,9	0,07
REPAIR		BER	***Smug1***	nd		**-6,0**	**0,003**	**-5,9**	**0,002**
REPAIR		BER	***Tdg***	1,9	0,09	-2,8	0,11	**-4,9**	**0,02**
REPAIR		BER	***Ung***	**2,6**	**0,02**	1,5	0,08	1,4	0,11
REPAIR		BER	***Apex1***	1,4	0,09	**-2,5**	**0,02**	**-2,4**	**0,002**
REPAIR		BER	***Parp1***	**2,3**	**0,04**	**-2,2**	**0,04**	-1,5	0,2
REPAIR		BER	***Parp2***	**-1,7**	**0,02**	**-3,7**	**0,007**	-2,1	0,08
REPAIR		BER	***Lig3***	nd		**-2,3**	**0,01**	**-3,2**	**0,02**
REPAIR		BER	***Xrcc1***	-1,2	0,67	**-3,0**	**0,009**	**-2,6**	**0,03**
REPAIR		[illegible]	***Xpa***	-1,3	0,14	**-3,5**	**0,01**	**-3,8**	**0,04**
REPAIR		[illegible]	***Xpc***	-1,4	0,21	**-4,4**	**0,008**	**-7,5**	**0,02**

SSB	NE	***Rad23a***	-1,2	0,58	**-2,9**	**0,03**	**-2,2**	**0,04**
		Ercc1	1,3	0,39	**-3,6**	**0,007**	-2,1	0,09
		Pold	**1,7**	**0,03**	**-2,2**	**0,01**	-1,3	0,31
	MMR	***Msh2***	1,4	0,20	**-2,7**	**0,01**	-1,8	0,10
		Msh3	1,2	0,39	**-4,2**	**0,005**	**-2,6**	**0,04**
		Mlh1	1,4	0,66	-1,7	0,08	-1,2	0,45
		Mlh3	-1,1	0,98	**-4,3**	**0,01**	**-3,7**	**0,046**
		Pms1	1,1	0,73	-2,3	**0,03**	-1,5	0,24
		Pms2	1,2	0,36	**-2,5**	**0,003**	**-3,0**	**0,04**
		Trex1	-1,0	0,85	-2,1	0,13	**-3,0**	**0,004**
	DDR	***Mgmt***	-3,0	0,15	**-3,6**	**0,04**	-1,7	0,28

Statistical significance was set at $p < 0,05$ and shown in bold. Up-regulation is shown in red and down-regulation in blue. nd indicates that the gene was not tested in the indicated condition.
