# Supplementary material for: CD8 Memory Cells Develop Unique DNA Repair Mechanisms Favoring Productive Division
Source: PLoS One. 2015 Oct 20;10(10):e0140849. doi: 10.1371/journal.pone.0140849 (PMC4613136; doi:10.1371/journal.pone.0140849)
Supplement: S2 Table — (PDF) [file pone.0140849.s002.pdf]

Table S2. Contraction phase of primary CD8 responses without and with CD4 help

Category	Function	Group	Gene	Contraction phase (d19)			
				Without CD4 help		With CD4 help	
				Fold change	p value	Fold change	p value
DSB DETECTION	SENSORS		*H2ax*	**3,1**	**0,003**	1,6	0,16
DSB DETECTION	SENSORS	MRN	*Mre11*	1,1	0,75	-1,3	0,13
DSB DETECTION	SENSORS	MRN	*Rad50*	1,3	0,42	1,1	0,36
DSB DETECTION	SENSORS		*Atm*	-1,1	0,97	-1,3	0,07
DSB DETECTION	MEDIATOR		*Brca1*	2,0	0,09	2,6	0,27
DSB DETECTION	EFFECTOR		*Tp53*	-1,2	0,16	**-1,5**	**0,04**
SSB DETECTION	SENSORS		*H2ax*	**3,1**	**0,003**	1,6	0,16
SSB DETECTION	SENSORS	9-1-1	*Rad9a*	1,1	0,72	-1,2	0,43
SSB DETECTION	SENSORS	9-1-1	*Rad9b*	**-2,4**	**0,0006**	**-2,3**	**0,02**
SSB DETECTION	SENSORS	9-1-1	*Rad1*	1,1	0,72	-1,2	0,33
SSB DETECTION	SENSORS	9-1-1	*Hus1*	1,5	0,18	1,1	1,08
SSB DETECTION	SENSORS		*Rad17*	1,1	0,73	-1,1	0,52
SSB DETECTION	MEDIATOR		*Brca1*	2,0	0,09	2,6	0,27
SSB DETECTION	TRANSDUCER		*Chk1*	3,06	0,06	1,94	0,16
DSB REPAIR		NHEJ	*Xrcc6*	-1,1	0,52	-1,5	0,14
DSB REPAIR		NHEJ	*Prkdc*	1,2	0,40	-1,1	0,52
DSB REPAIR		HR	*Rad52*	1,2	0,59	-1,5	0,16
DSB REPAIR		HR	*Rad51*	3,9	0,06	**3,9**	**0,03**
DSB REPAIR		HR	*Rad51b*	1,9	0,06	1,4	0,44
DSB REPAIR		HR	*Rad51c*	1,7	0,11	1,5	0,41
DSB REPAIR		HR	*Xrcc2*	1,3	0,21	1,5	0,15
DSB REPAIR		HR	*Xrcc3*	-2,1	0,23	**-2,0**	**0,008**
DSB REPAIR		HR	*Rad54*	1,1	0,63	-1,0	0,85
DSB REPAIR		HR	*Brca2*	1,4	0,46	1,4	0,49
DSB REPAIR		HR	*Pold*	**1,5**	**0,02**	1,2	0,51
DSB REPAIR		HR	*Lig1*	**3,4**	**0,02**	1,8	0,09
SSB REPAIR		BER	*Mpg*	1,3	0,43	1,1	0,76
SSB REPAIR		BER	*Ogg1*	1,2	0,44	-1,2	0,42
SSB REPAIR		BER	*Tdg*	1,1	0,73	-1,1	0,77
SSB REPAIR		BER	*Ung*	1,2	0,51	1,1	0,69
SSB REPAIR		BER	*Apex1*	-1,1	0,38	-1,3	0,08
SSB REPAIR		BER	*Parp1*	**1,5**	**0,04**	1,2	0,45
SSB REPAIR		BER	*Parp2*	-1,3	0,22	**-1,7**	**0,02**
SSB REPAIR		BER	*Xrcc1*	-1,2	0,69	-1,3	0,14
SSB REPAIR		NER	*Xpa*	1,2	0,52	1,0	0,84
SSB REPAIR		NER	*Xpc*	-1,2	0,56	-1,1	0,62
SSB REPAIR		NER	*Rad23a*	1,1	0,7	-1,0	0,89
SSB REPAIR		NER	*Ercc1*	1,3	0,33	-1,1	0,5
SSB REPAIR		NER	*Pold*	**1,5**	**0,02**	1,2	0,51
SSB REPAIR		MMR	*Msh2*	**1,5**	**0,05**	1,1	0,63
SSB REPAIR		MMR	*Msh3*	1,3	0,41	-1,2	0,23
SSB REPAIR		MMR	*Mlh1*	-1,3	0,94	-1,3	0,55
SSB REPAIR		MMR	*Mlh3*	-1,2	0,5	**-2,1**	**0,008**
SSB REPAIR		MMR	*Pms1*	-1,0	0,96	-1,2	0,33
SSB REPAIR		MMR	*Pms2*	0,4	1,22	1,0	0,99
SSB REPAIR		MMR	*Trex1*	1,2	0,48	1,4	0,25
SSB REPAIR		DDR	*Mgmt*	-4,6	0,06	**-12,2**	**0,04**

Statistical significance was set at $p < 0,05$ and shown in bold.
Up-regulation is shown in red and down-regulation in blue.
