# Supplementary material for: CD8 Memory Cells Develop Unique DNA Repair Mechanisms Favoring Productive Division
Source: PLoS One. 2015 Oct 20;10(10):e0140849. doi: 10.1371/journal.pone.0140849 (PMC4613136; doi:10.1371/journal.pone.0140849)
Supplement: S3 Table — (PDF) [file pone.0140849.s003.pdf]

Table S3. Memory phase without and with CD4 help

Category	Subgroup	Pathway	Gene	Memory phase: Without CD4 help – Fold change	Without CD4 help – p value	With CD4 help – Fold change	With CD4 help – p value
DSB DETECTION	SENSORS		*H2ax*	**3,0**	**0,001**	1,7	0,08
DSB DETECTION	SENSORS	MRN	*Mre11*	1,8	0,06	-1,4	0,34
DSB DETECTION	SENSORS	MRN	*Rad50*	1,1	0,38	1,0	0,73
DSB DETECTION	SENSORS		*Atm*	**-3,0**	**0,001**	-1,4	0,12
DSB DETECTION	MEDIATOR		*Brca1*	1,1	0,64	1,2	0,63
DSB DETECTION	EFFECTOR		*Tp53*	1,0	0,1	**-1,5**	**0,03**
SSB DETECTION	SENSORS		*H2ax*	**3,0**	**0,001**	1,7	0,08
SSB DETECTION	SENSORS	9-1-1	*Rad9a*	**-2,1**	**0,01**	-2,3	0,06
SSB DETECTION	SENSORS	9-1-1	*Rad9b*	nd		**-2,8**	**0,04**
SSB DETECTION	SENSORS	9-1-1	*Rad1*	-1,1	0,75	-1,4	0,12
SSB DETECTION	SENSORS	9-1-1	*Hus1*	1,1	0,78	-1,1	0,81
SSB DETECTION	SENSORS		*Rad17*	**-5,0**	**0,0008**	-1,4	0,13
SSB DETECTION	SENSORS		*Atr*	**-4,21**	**0,02**	-1,34	0,29
SSB DETECTION	MEDIATOR		*Brca1*	1,1	0,64	1,2	0,63
SSB DETECTION	TRANSDUCER		*Chk1*	1,39	0,33	1,82	0,18
DSB REPAIR		NHEJ	*Xrcc5*	1,5	0,34	1,23	0,28
DSB REPAIR		NHEJ	*Xrcc6*	-1,2	0,53	-2,0	0,06
DSB REPAIR		NHEJ	*Prkdc*	**-4,6**	**0,02**	-1,4	0,41
DSB REPAIR		NHEJ	*Xrcc4*	-1,23	0,45	-1,11	0,21
DSB REPAIR		NHEJ	*Lig4*	-2,14	0,05	-1,72	0,18
DSB REPAIR		HR	*Rad52*	1,1	0,69	-1,5	0,32
DSB REPAIR		HR	*Rad51*	**3,3**	**0,02**	1,9	0,29
DSB REPAIR		HR	*Rad51b*	-1,0	0,87	1,6	0,26
DSB REPAIR		HR	*Rad51c*	**-1,8**	**0,044**	-1,2	0,44
DSB REPAIR		HR	*Rad51d*	-2,46	0,05	-1,49	0,06
DSB REPAIR		HR	*Xrcc2*	**3,5**	**0,004**	1,4	0,17
DSB REPAIR		HR	*Xrcc3*	-1,4	0,37	**-4,2**	**0,02**
DSB REPAIR		HR	*Rpa*	**-3,69**	**0,03**	1,03	0,76
DSB REPAIR		HR	*Rad54*	**-3,8**	**0,04**	-1,0	0,95
DSB REPAIR		HR	*Brca2*	-1,1	0,95	-1,1	0,86
DSB REPAIR		HR	*Pold*	-1,4	0,23	-1,3	0,66
DSB REPAIR		HR	*Lig1*	**3,0**	**0,02**	1,5	0,2
SSB REPAIR		BER	*Mpg*	-1,0	0,87	-1,5	0,39
SSB REPAIR		BER	*Ogg1*	1,0	0,87	-1,3	0,56
SSB REPAIR		BER	*Smug1*	1,31	0,43	-1,15	0,54
SSB REPAIR		BER	*Tdg*	**-18,3**	**0,03**	**-2,2**	**0,04**
SSB REPAIR		BER	*Ung*	**2,0**	**0,04**	-1,4	0,2
SSB REPAIR		BER	*Apex1*	**-2,3**	**0,03**	-1,4	0,24
SSB REPAIR		BER	*Parp1*	-1,3	0,28	-1,3	0,49
SSB REPAIR		BER	*Parp2*	-1,2	0,51	-2,2	0,06
SSB REPAIR		BER	*Lig3*	**-3,59**	**0,01**	-1,51	0,1
SSB REPAIR		BER	*Xrcc1*	1,2	0,31	-1,9	0,08
SSB REPAIR		NER	*Xpa*	**1,9**	**0,005**	1,1	0,6
SSB REPAIR		NER	*Xpc*	-1,9	0,01	-1,3	0,59
SSB REPAIR		NER	*Rad23a*	-1,3	0,41	-1,6	0,18
SSB REPAIR		NER	*Ercc1*	1,3	0,13	-1,3	0,34
SSB REPAIR		NER	*Pold*	-1,4	0,23	-1,3	0,66
SSB REPAIR		MMR	*Msh2*	**-1,8**	**0,01**	1,2	0,37
SSB REPAIR		MMR	*Msh3*	1,1	0,71	-1,4	0,32
SSB REPAIR		MMR	*Mlh1*	-4,5	0,11	-1,7	0,29
SSB REPAIR		MMR	*Mlh3*	**-2,1**	**0,02**	**-2,5**	**0,02**
SSB REPAIR		MMR	*Pms1*	-1,6	0,24	-1,5	0,2
SSB REPAIR		MMR	*Pms2*	-2,8	0,06	-1,2	0,59
SSB REPAIR		MMR	*Trex1*	**-10,4**	**0,002**	**-4,7**	**0,004**
SSB REPAIR		DDR	*Mgmt*	**-3,8**	**0,01**	**-15,5**	**0,04**

Statistical significance was set at p < 0,05 and shown in bold. Up-regulation is shown in red and down-regulation in blue. nd indicates that the gene was not tested in the indicated condition.
