# Supplementary material for: CD8 Memory Cells Develop Unique DNA Repair Mechanisms Favoring Productive Division
Source: PLoS One. 2015 Oct 20;10(10):e0140849. doi: 10.1371/journal.pone.0140849 (PMC4613136; doi:10.1371/journal.pone.0140849)
Supplement: S4 Table — (PDF) [file pone.0140849.s004.pdf]

Table S4. Additional modified genes with secondary roles in DNA damage response

	Gene	Without CD4 help					
		Primary response		Secondary response			
		d6		d4		d6	
		Fold change	p value	Fold change	p value	Fold change	p value
DNA BINDING	*Trpc2*	-2,8	0,001	nd		nd	
	Chaf1a	10,0	0,003	nd		nd	
	Terf1	-1,1	0,72	-2,52	0,01	-34,74	0,003
CELL CYCLE ARREST AND APOPTOSIS	*Cdk7*	nd		-4,78	0,008	-5,6	0,02
	Ddit3	nd		-2,18	0,03	-2,82	0,01
	Gadd45a	1,02	0,91	-1,9	0,004	-2,04	0,1
	Ppm1d	nd		-1,72	0,03	-1,13	0,76
	Abl	nd		-1,3	0,5	-2,52	0,01
	Cdkn1a	nd		2,0	0,1	7,1	0,02
DSB REPAIR	*Brip1*	nd		4,92	0,0007	11,28	0,0001
	Mdc1	nd		-3,72	0,02	-2,44	0,06
	Rad21	1,12	0,63	-2,45	0,03	-1,2	0,45
	Wrn	-1,13	0,74	-2,06	0,02	-1,44	0,46
BER	*Apex2*	nd		-2,93	0,06	-1,58	0,04
	Fen1	3,6	0,01	1,42	0,55	2,98	0,001
	Mutyh	1,02	0,95	-3,29	0,006	-1,43	0,29
	Neil1	nd		-4,94	0,003	-3,96	0,04
	Neil2	nd		-5,43	0,02	-5,64	0,04
	Neil3	nd		7,45	0,00007	21,06	0,00002
	Nthl1	-1,12	0,79	-3,81	0,01	-4,58	0,003
	Parp3	nd		-4,53	0,01	-3,83	0,04
	Polb	nd		-2,6	0,002	-2,25	0,03
NER	*Atxn3*	nd		-3,56	0,007	-3,14	0,044
	Ddb1	nd		-3,1	0,02	-1,85	0,12
	Ddb2	nd		-4,1	0,003	-3,22	0,04
	Ercc2	nd		-3,8	0,02	-2,8	0,11
	Ercc4	nd		-3,56	0,01	-3,47	0,03
	Ercc5	nd		-4,62	0,004	-3,41	0,047
	Ercc6	nd		-4,23	0,009	-3,44	0,02
	Ercc8	nd		-3,6	0,02	-2,8	0,06
	Mms19	nd		-4,62	0,007	-3,04	0,06
	Pole	10,0	0,002	2,84	0,008	4,1	0,06
	Poll	nd		-3,2	0,007	-2,51	0,08
	Slk	-1,28	0,2	-2,76	0,009	-2,38	0,006
	Xab2	nd		-3,26	0,005	-3,24	0,03
MMR	*Exo1*	22,3	0,01	3,88	0,0007	10,6	0,001

	Gene	Secondary responses							
		Without CD4 help				With CD4 help			
		d4		d6		d4		d6	
		Fold change	p value	Fold change	p value	Fold change	p value	Fold change	p value
DNA BINDING	*Chaf1a*	nd		nd		11,4	0,02	5,41	0,006
	Terf1	-2,52	0,01	-34,74	0,003	-1,62	0,11	-1,74	0,08
CELL CYCLE ARREST AND APOPTOSIS	*Cdk7*	-4,78	0,008	-5,6	0,02	-3,8	0,04	nd	
	Ddit3	-2,18	0,03	-2,82	0,01	nd		nd	
	Gadd45a	-1,9	0,004	-2,04	0,1	-1,6	0,014	-3,47	0,006
	Ppm1d	-1,72	0,03	-1,13	0,76	nd		nd	
	Abl	-1,3	0,5	-2,52	0,01	nd		nd	
	Cdkn1a	2,0	0,1	7,1	0,02	nd		nd	
DSB REPAIR	*Brip1*	4,92	0,0007	11,28	0,0001	9,1	0,002	nd	
	Mdc1	-3,72	0,02	-2,44	0,06	nd		nd	
	Rad21	-2,45	0,03	-1,2	0,45	1,6	0,021	1,23	0,51
	Wrn	-2,06	0,02	-1,44	0,46	-1,6	0,046	-1,65	0,02
BER	*Apex2*	-2,93	0,06	-1,58	0,04	1,07	0,91	nd	
	Fen1	1,42	0,55	2,98	0,001	4,4	0,009	3,32	0,07
	Mutyh	-3,29	0,006	-1,43	0,29	1,42	0,48	-1,25	0,51
	Neil1	-4,94	0,003	-3,96	0,04	-2,12	0,14	nd	
	Neil2	-5,43	0,02	-5,64	0,04	-3,9	0,02	nd	
	Neil3	7,45	0,00007	21,06	0,00002	12,8	0,01	nd	
	Nthl1	-3,81	0,01	-4,58	0,003	-2,29	0,08	-2,33	0,22

	Gene	Fold change	p value	Fold change	p value	Fold change	p value	Fold change	p value
	Parp3	**-4,53**	**0,01**	**-3,83**	**0,04**	**-3,1**	**0,03**	nd	
	Polb	**-2,6**	**0,002**	**-2,25**	**0,03**	-1,23	0,96	nd	
NER	*Atxn3*	**-3,56**	**0,007**	**-3,14**	**0,044**	-1,96	0,13	nd	
	Ddb1	**-3,1**	**0,02**	-1,85	0,12	1,05	0,82	nd	
	Ddb2	**-4,1**	**0,003**	**-3,22**	**0,04**	-2,22	0,14	nd	
	Ercc2	**-3,8**	**0,02**	-2,8	0,11	-1,92	0,16	nd	
	Ercc4	**-3,56**	**0,01**	**-3,47**	**0,03**	-2,31	0,12	nd	
	Ercc5	**-4,62**	**0,004**	**-3,41**	**0,047**	-2,45	0,1	nd	
	Ercc6	**-4,23**	**0,009**	**-3,44**	**0,02**	-2,79	0,1	nd	
	Ercc8	**-3,6**	**0,02**	-2,8	0,06	-1,96	0,16	nd	
	Gtf2h1	nd		nd		-1,2	0,36	**-2,43**	**0,02**
	Mms19	**-4,62**	**0,007**	-3,04	0,06	-1,61	0,42	nd	
	Pole	**2,84**	**0,008**	4,1	0,06	**3,7**	**0,02**	**2,47**	**0,01**
	Poll	**-3,2**	**0,007**	-2,51	0,08	-1,08	0,7	nd	
	Rad23b	**-2,56**	**0,02**	**-2,08**	**0,01**	**-1,8**	**0,04**	nd	
	Slk	**-2,76**	**0,009**	**-2,38**	**0,006**	-1,58	0,12	-1,59	0,1
	Xab2	**-3,26**	**0,005**	**-3,24**	**0,03**	**-1,7**	**0,03**	nd	
MMR	*Exo1*	**3,88**	**0,0007**	**10,6**	**0,001**	**10,9**	**0,0008**	**6,2**	**0,01**

	Gene	Contraction phase (d19) of primary response			
		Without CD4 help		With CD4 help	
		Fold change	p value	Fold change	p value
DNA BINDING	*Trpc2*	**-2,8**	**0,11**	**-3,45**	**0,0003**
NER	*Chaf1a*	**2,4**	**0,02**	1,96	0,11
	Pole	**3,8**	**0,045**	**2,19**	**0,02**

	Gene	Memory phase			
		Without CD4 help		With CD4 help	
		Fold change	p value	Fold change	p value
CELL CYCLE	*Cdk7*	**-3,5**	**0,001**	-1,63	0,2
	Gadd45a	-1,05	0,75	**1,29**	**0,048**
DSB	*Mif*	**-4,7**	**0,02**	1,04	0,76
	Brip1	**-7,1**	**0,001**	-1,35	0,35
BER	*Wrn*	**-5,7**	**0,004**	-1,37	0,34
	Fen1	**-3,2**	**0,01**	-2,1	0,09
	Mutyh	**-1,9**	**0,04**	-1,82	0,17
	Neil1	**-2,2**	**0,04**	-1,58	0,31
	Nthl1	**-8,9**	**0,02**	-2,29	0,21
	Parp3	**-1,9**	**0,03**	-1,45	0,14
NER	*Polb*	**-7,4**	**0,02**	**-9,15**	**0,001**
	Atxn3	**-1,7**	**0,02**	-1,05	0,85
	Dbd2	**-3,2**	**0,02**	-1,97	0,11
	Ercc2	**-2,0**	**0,04**	-1,22	0,38
	Ercc3	**-6,5**	**0,002**	-1,1	0,7
	Ercc5	**-2,3**	**0,02**	-1,21	0,59
	Mms19	**1,6**	**0,03**	-1,37	0,48
MMR	*Rad23b*	**68,5**	**0,000008**	-1,25	0,47
	Msh5	**-10,3**	**0,006**	**-3,56**	**0,03**
	Msh6	nd		**-2,96**	**0,04**

	Gene	PMEF	
		Fold change	p value
CELL CYCLE	*Ddit3*	**-1,4**	**0,02**
DSB	*Gadd45a*	**-1,7**	**0,02**
BER	*Brip1*	**1,4**	**0,03**
	Apex2	**1,6**	**0,004**
	Fen1	**2,2**	**0,01**
	Mutyh	**1,3**	**0,04**
NER	*Neil3*	**1,6**	**0,009**
	Ercc3	**1,7**	**0,02**
	Pole	**1,4**	**0,04**
	Rad23b	**-1,2**	**0,04**
	Slk	**1,3**	**0,048**
	Xab2	**-1,2**	**0,009**

Statistical significance was set at $p < 0,05$ and shown in bold. Up-regulation is shown in red and down-regulation in blue. nd indicates that the gene was not tested in the indicated condition. Among the most differentially expressed genes we mentioned:

Chaf1a: mediates chromatin assembly in DNA replication and repair. *Terf1*: is involved in the regulation of telomere length and protection. *Neil1/2/3*: DNA glycosylases initiating the first step in base excision repair. *Brip1*: associates with BRCA1 in HR. *Exo1*: an endonuclease acting in MMR. *Cdkn1a*: a cycline inhibitor, blocks cell proliferation upon DNA damge and may induce apoptosis (www.genecards.org).
