# Supplementary material for: CD8 Memory Cells Develop Unique DNA Repair Mechanisms Favoring Productive Division
Source: PLoS One. 2015 Oct 20;10(10):e0140849. doi: 10.1371/journal.pone.0140849 (PMC4613136; doi:10.1371/journal.pone.0140849)
Supplement: S5 Table — (PDF) [file pone.0140849.s005.pdf]

Table S5. Additional modified genes with less defined function in DNA damage response

Gene	Without CD4 help					
	Primary response		Secondary response			
	d6		d4		d6	
	Fold change	p value	Fold change	p value	Fold change	p value
Fancd2	nd		**7,2**	**0,0007**	**32,97**	**0,03**
Poli	-1,59	0,17	-4,25	0,06	**-2,85**	**0,005**
Polh	**1,9**	**0,03**	-1,56	0,06	1,62	0,17
Pttg1	nd		**-2,43**	**0,01**	-1,52	0,45
Rad18	1,16	0,36	-2,36	0,07	**-2,14**	**0,04**
Rbbp4	**-1,7**	**0,03**	nd		nd	
Rbm4	**-2,0**	**0,02**	nd		nd	
Rev1	**-1,9**	**0,03**	**-3,38**	**0,006**	**-2,11**	**0,03**
Rfc1	nd		**-1,57**	**0,03**	-1,28	0,27
Sumo	-1,04	0,77	**-1,39**	**0,04**	1,09	0,63
Top3a	nd		**-3,68**	**0,008**	**-3,52**	**0,04**
Top3b	nd		**-3,51**	**0,0008**	**-3,69**	**0,01**
Xrcc6bp1	nd		**-2,12**	**0,048**	**-2,46**	**0,01**

Gene	Secondary responses							
	Without CD4 help				With CD4 help			
	d4		d6		d4		d6	
	Fold change	p value	Fold change	p value	Fold change	p value	Fold change	p value
Fancd2	**7,2**	**0,0007**	**32,97**	**0,03**	nd		nd	
Polh	-1,56	0,06	1,62	0,17	1,8	0,22	1,14	0,7
Poli	-4,25	0,06	**-2,85**	**0,005**	-2,39	0,07	-3,38	0,1
Polk	nd		nd		**-1,9**	**0,01**	**-1,8**	**0,03**
Pttg1	**-2,43**	**0,01**	-1,52	0,45	-2,63	0,11	-1,89	0,23
Rad18	-2,36	0,07	**-2,14**	**0,04**	1,03	0,95	-1,28	0,32
Rbm4	nd		nd		**-2,4**	**0,03**	-2,46	0,06
Rev1	**-3,38**	**0,006**	**-2,11**	**0,03**	**-2,7**	**0,04**	-3	0,08
Rbbp4	nd		nd		-1,78	0,11	-1,82	0,16
Rfc1	**-1,57**	**0,03**	-1,28	0,27	1,1	0,8	nd	
Sumo	**-1,39**	**0,04**	1,09	0,63	-1,3	0,56	**-1,62**	**0,02**
Top3a	**-3,68**	**0,008**	**-3,52**	**0,04**	**-1,6**	**0,006**	nd	
Top3b	**-3,51**	**0,0008**	**-3,69**	**0,01**	-1,36	0,37	nd	
Ube2a	nd		nd		**-1,8**	**0,009**	**-1,75**	**0,007**
Xrcc6bp1	**-2,12**	**0,048**	**-2,46**	**0,01**	1,02	0,88	nd	

Gene	Contraction phase (d19) of primary response			
	Without CD4 help		With CD4 help	
	Fold change	p value	Fold change	p value
Polh	**1,7**	**0,04**	1,29	0,4
Poli	-1,2	0,67	**-1,93**	**0,02**
Rbm4	-1,5	0,27	**-1,76**	**0,02**
Xrn2	1,6	0,1	**1,57**	**0,009**

Gene	Memory phase			
	Without CD4 help		With CD4 help	
	Fold change	p value	Fold change	p value
Fancc	**-1,7**	**0,02**	-2,0	0,1
Fancg	**9,9**	**0,0001**	-2,11	0,38
Polh	**2,0**	**0,04**	1,1	0,64
Pttg1	**1,7**	**0,02**	-1,57	0,18
Rad18	**3,5**	**0,004**	1,34	0,17
Rbm4	**-5,3**	**0,008**	-1,1	0,73
Rev1	**3,6**	**0,0009**	1,16	0,06
Rfc1	**1,5**	**0,048**	1,06	0,73
Top3b	-1,68	0,19	**-1,51**	**0,02**

Statistical significance was set at $p < 0,05$ and shown in bold. Up-regulation is shown in red and down-regulation in blue. nd indicates that the gene was not tested in the indicated condition.
