# Supplementary material for: CD8 Memory Cells Develop Unique DNA Repair Mechanisms Favoring Productive Division
Source: PLoS One. 2015 Oct 20;10(10):e0140849. doi: 10.1371/journal.pone.0140849 (PMC4613136; doi:10.1371/journal.pone.0140849)
Supplement: S6 Table — (PDF) [file pone.0140849.s006.pdf]

Table S6. Secondary CD8 responses without and with CD4 help

			Gene	Without CD4 help				With CD4 help			
				d4		d6		d4		d6	
				Fold change	p value	Fold change	p value	Fold change	p value	Fold change	p value
DSB DETECTION	SENSORS		*H2ax*	1,7	0,02	2,2	0,006	3,0	0,03	2,02	0,12
		MRN	*Mre11*	-2,7	0,02	-1,8	0,1	-1,8	0,16	-2,59	0,09
			Rad50	-3,3	0,01	-2,1	0,05	-1,2	0,37	-1,49	0,26
			Nbn	-1,3	0,64	1,4	0,39	-1,5	0,70	nd	
			Atm	-4,2	0,004	-2,9	0,04	-1,8	0,11	-2,26	0,08
	MEDIATORS		*Tp53bp1*	-2,0	0,02	-1,9	0,02	nd		nd	
			Brca1	2,2	0,03	4,6	0,00007	7,7	0,003	5,22	0,01
	TRANSDUCER		*Chk2*	1,1	0,67	4,5	0,07	3,9	0,33	nd	
	EFFECTOR		*Tp53*	-2,7	0,03	-1,9	0,008	-1,8	0,02	-2,26	0,03
SSB DETECTION	SENSORS		*H2ax*	1,7	0,02	2,2	0,006	3,0	0,03	2,02	0,12
		9-1-1	*Rad9a*	-2,7	0,01	-2,9	0,00007	-1,4	0,3	-1,38	0,35
			Rad9b	nd		nd		-2,8	0,09	-2,5	0,2
			Rad1	-2,5	0,03	-2,9	0,00002	-1,9	0,06	-2,08	0,06
			Hus1	-1,8	0,1	-1,1	0,95	-1,1	0,73	-2,23	0,09
			Rad17	-3,1	0,08	-1,9	0,001	-1,4	0,31	-1,64	0,06
			Atr	-3,4	0,04	-3,1	0,08	-1,6	0,34	nd	
	MEDIATOR		*Brca1*	2,2	0,03	4,6	0,00007	7,7	0,003	5,22	0,01
	TRANSDUCER		*Chk1*	6,6	0,001	17,3	0,01	9,0	0,02	5,28	0,01
DSB REPAIR		NHEJ	*Xrcc5*	-2,7	0,02	-3,5	0,012	-1,5	0,07	nd	
			Xrcc6	-2,2	0,02	-2,2	0,06	-1,0	0,96	nd	
			Prkdc	-3,0	0,01	-2,8	0,02	-2,0	0,09	-3,23	0,19
			Xrcc4	-4,1	0,003	-5,7	0,009	-2,6	0,0004	nd	
			Lig4	-2,9	0,009	-2,0	0,03	-1,7	0,28	nd	
		HR	*Rad52*	-4,8	0,009	-4,4	0,02	-2,7	0,02	-2,24	0,06
			Rad51	4,2	0,0002	16,8	0,04	10,8	0,002	13,25	0,006
			Rad51b	1,3	0,65	2,4	0,007	4,4	0,01	2,82	0,008
			Rad51c	1,7	0,09	7,2	0,00005	3,4	0,001	1,97	0,046
			Rad51d	-5,4	0,001	-3,2	0,02	-3,5	0,03	nd	
			Xrcc2	-1,8	0,01	-1,2	0,32	1,7	0,006	4,19	0,007
			Xrcc3	-3,2	0,02	-2,9	0,04	-1,9	0,03	1,25	0,1
			Rpa	-2,2	0,05	-1,4	0,25	1,4	0,26	nd	
			Rad54	3,5	0,003	6,8	0,0005	10,4	0,00002	-1,86	0,19
			Brca2	-1,1	0,8	1,9	0,1	4,8	0,001	2,11	0,13
			Pold	-2,2	0,01	-1,3	0,31	1,6	0,21	1,01	0,91
			Lig1	1,9	0,046	5,4	0,0006	8,8	0,004	5,17	0,0004
SSB REPAIR		BER	*Mpg*	-2,0	0,06	-2,1	0,05	-1,0	0,96	-1,28	0,49
			Ogg1	-3,7	0,02	-2,9	0,07	-1,3	0,49	-1,91	0,19
			Smug1	-6,0	0,003	-5,9	0,002	-2,5	0,04	nd	
			Tdg	-2,8	0,11	-4,9	0,02	2,2	0,005	1,62	0,11
			Ung	1,5	0,08	1,4	0,11	2,8	0,04	1,89	0,003
			Apex1	-2,5	0,02	-2,4	0,002	-1,0	0,91	-2,37	0,15
			Parp1	-2,2	0,04	-1,5	0,2	1,4	0,37	-1,31	0,42
			Parp2	-3,7	0,007	-2,1	0,08	-1,0	0,68	-1,57	0,32
			Lig3	-2,3	0,01	-3,2	0,02	-1,4	0,41	nd	
			Xrcc1	-3,0	0,009	-2,6	0,03	-1,2	0,35	-1,13	0,63
		NER	*Xpa*	-3,5	0,01	-3,8	0,04	-2,7	0,006	-2,14	0,07
			Xpc	-4,4	0,008	-7,5	0,02	-5,0	0,0006	-4,47	0,06
			Rad23a	-2,9	0,03	-2,2	0,04	-1,0	0,82	-1,46	0,3
			Ercc1	-3,6	0,007	-2,1	0,09	-1,2	0,64	-2,06	0,14
			Pold	-2,2	0,01	-1,3	0,31	1,6	0,21	1,01	0,91
			Msh2	-2,7	0,01	-1,8	0,10	-1,1	0,97	-2,17	0,03

MMR	***Msh3***	**-4,2**	**0,005**	**-2,6**	**0,04**	-1,5	0,36	-2,17	0,09
	Mlh1	-1,7	0,08	-1,2	0,45	1,7	0,25	-1,35	0,24
	Mlh3	**-4,3**	**0,01**	**-3,7**	**0,046**	-2,0	0,19	-2,76	0,11
	Pms1	-2,3	**0,03**	-1,5	0,24	-1,1	0,82	-1,26	0,35
	Pms2	**-2,5**	**0,003**	**-3,0**	**0,04**	-1,1	0,9	-1,29	0,34
	Trex1	-2,1	0,13	**-3,0**	**0,004**	**-2,0**	**0,03**	3,06	0,5
DDR	***Mgmt***	**-3,6**	**0,04**	-1,7	0,28	-1,5	0,42	**-3,35**	**0,046**

Statistical significance was set at $p < 0,05$ and shown in bold. Up-regulation is shown in red and down-regulation in blue. nd indicates that the gene was not tested in the indicated condition.
