# Supplementary material for: CD8 Memory Cells Develop Unique DNA Repair Mechanisms Favoring Productive Division
Source: PLoS One. 2015 Oct 20;10(10):e0140849. doi: 10.1371/journal.pone.0140849 (PMC4613136; doi:10.1371/journal.pone.0140849)
Supplement: S7 Table — (PDF) [file pone.0140849.s007.pdf]

Table S7. Exponential growing *vs.* resting PMEF cells

Pathway	Function	Complex	Gene	PMEF Fold change	PMEF p value
DSB DETECTION	SENSORS		*H2ax*	1,0	0,97
		MRN	*Mre11*	-1,1	0,75
			Rad50	1,1	0,86
			Nbn	1,22	0,34
			Atm	-1,1	0,77
	MEDIATORS		*Tp53bp1*	1,08	0,72
			Brca1	1,4	0,25
	TRANSDUCER		*Chk2*	1,26	0,48
	EFFECTOR		*Tp53*	-1,2	0,26
SSB DETECTION	SENSORS		*H2ax*	1,0	0,97
		9-1-1	*Rad9a*	1,2	0,21
			Rad1	-1,0	0,97
			Hus1	-1,1	0,48
			Rad17	-1,2	0,33
			Atr	1,56	0,21
	MEDIATOR		*Brca1*	1,4	0,25
	TRANSDUCER		*Chk1*	1,27	0,2
DSB REPAIR		NHEJ	*Xrcc5*	**-1,36**	**0,02**
			Xrcc6	-1,2	0,33
			Prkdc	1,4	0,18
			Xrcc4	-1,1	0,5
			Lig4	**1,34**	**0,02**
		HR	*Rad52*	-1,2	0,17
			Rad51	1,3	0,19
			Rad51b	-1,2	0,46
			Rad51c	-1,0	0,88
			Rad51d	1,2	0,32
			Xrcc2	1,1	0,5
			Xrcc3	1,3	0,19
			Rpa	-1,09	0,79
			Rad54	1,6	0,07
			Brca2	1,2	0,84
			Pold	1,1	0,52
			Lig1	-1,1	0,65
SSB REPAIR		BER	*Mpg*	-1,1	0,79
			Ogg1	-1,2	0,39
			Smug1	-1,25	0,06
			Tdg	-1,2	0,06
			Ung	**1,3**	**0,04**
			Apex1	-1,1	0,5
			Parp1	-1,2	0,4
			Parp2	-1,5	0,33
			Lig3	1,22	0,09
			Xrcc1	-1,0	0,92
		NER	*Xpa*	-1,2	0,39
			Xpc	1,0	0,96
			Rad23a	-1,1	0,26
			Ercc1	-1,0	0,67
			Pold	1,1	0,52

	MMR	***Msh2***	1,0	0,76
		Msh3	-1,1	0,76
		Mlh1	-1,0	0,92
		Mlh3	-1,4	0,18
		Pms1	-1,2	0,24
		Pms2	1,2	0,4
		Trex1	1,9	0,08
	DDR	***Mgmt***	-1,1	0,38

Statistical significance was set at $p < 0,05$ and shown in bold. Up-regulation is shown in red and down-regulation in blue.
